# Exploring the West African forest island phenomenon: scientific insights gained, successes achieved and capacities strengthened

**DOI:** 10.1098/rsfs.2023.0078

**Published:** 2024-08-09

**Authors:** Vincent Logah, Jamiu O. Azeez, Halidou Compaore, Samuel Ayodele Mesele, Caleb Melenya Ocansey, Amelie B. Bougma, Erasmus Narteh Tetteh, Elmar Veenendaal, Jon Lloyd

**Affiliations:** ^1^ Department of Crop and Soil Sciences, Kwame Nkrumah University of Science and Technology, Kumasi, Ghana; ^2^ Federal University of Agriculture Abeokuta, Abeokuta, Nigeria; ^3^ Institut de l’Environnement et de Recherches Agricoles, Tougan, Burkina Faso; ^4^ International Institute of Tropical Agriculture (IITA), Headquarters Ibadan, Ibadan, Nigeria; ^5^ Institute of Environmental Sciences, Hungarian University of Agriculture and Life Sciences, Gödöllő, Hungary; ^6^ CSIR-Crops Research Institute, Fumesua, Ghana; ^7^ Plant Ecology and Nature Conservation Group, Wageningen University, 6700 AA Wageningen, The Netherlands; ^8^ Department of Life Sciences, Imperial College of Science and Technology, London, UK

**Keywords:** capacity strengthening, ecosystems, forest island, savanna, soil aggregates, soil nutrients

## Abstract

Anthropogenic activities around local villages in mesic savanna landscapes of West Africa have resulted in soil improvement and forest establishment outside their climatic zones. Such unique ‘forest islands’ have been reported to provide ecosystem services including biodiversity conservation. However, the science underpinning their formations is limitedly studied. In 2015 and with funding support from the Royal Society-DFID (now FCDO), we set out to investigate the biogeochemistry of the forest islands in comparison with adjacent natural savanna and farmlands across 11 locations in Burkina Faso, Ghana and Nigeria. Our results showed that the forest islands do not differ significantly from the adjoining ecosystems in soil mineralogy implying that their formation was anthropogenically driven. We observed greater soil organic carbon and nutrient distributions in the forest islands, which also had more stable macro (>500 μm) and meso-aggregates (500–250 μm) than the adjoining agricultural lands. We found that soil micro-aggregate (250–53 μm) stability was climate (precipitation) driven in the West African ecosystems while meso- and macro-aggregate stability was land-use driven. In one of the unique forest islands we studied in the Mole National Park of Ghana, we found its mineral-associated organic carbon over 40% greater than the adjoining natural savanna with potential implications for the achievement of the global initiative of the ‘4p1000’ in West Africa. We conclude that the North–South–South research collaboration has established clearly, the science underlying the age-long West African forest island phenomenon and has, among many successes, led to capacity building of young scientists driving cutting-edge research in climate change adaptation and food systems transformation in the sub-region.

## 1. Introduction

### 1.1. Background

Forest islands comprising dense woody vegetation with deciduous and evergreen tree species are unique landscape elements surrounding local villages in the mesic savannas of West Africa [1]. Early botanists considered forest islands as relics of larger forests destroyed by indiscriminate human activity [2–4]. However, more recently, they have been considered ‘presumably the direct outcome of human activity’ [5]. This notion has been supported by palaeo evidence showing that forest islands are cultivated deliberately on what were originally savanna soils [6]. Communal activities such as the deposition of organic materials including household wastes and animal faecal matter have led over time to soil amelioration. This together with tree species selection, managed exploitation and fire protection has culminated in the establishment of such forest islands, most of which are still under active management in West Africa. Rather than decrease in size, these vegetation formations tend to have expanded in recent times [7–9] with a possible role in carbon sequestration [10]. This unique phenomenon contradicts the dominant perspective in the scientific literature that people only degrade natural soils [11]. The formation of forest islands in the savanna landscapes of West Africa is similar to the Amazonian dark earth or terra preta phenomenon [12,13] with major implications for soil quality. The soils of the unique West African forest islands have been described as ‘African dark earths’ [8].

### 1.2. Conceptual framework

**Figure 1.**
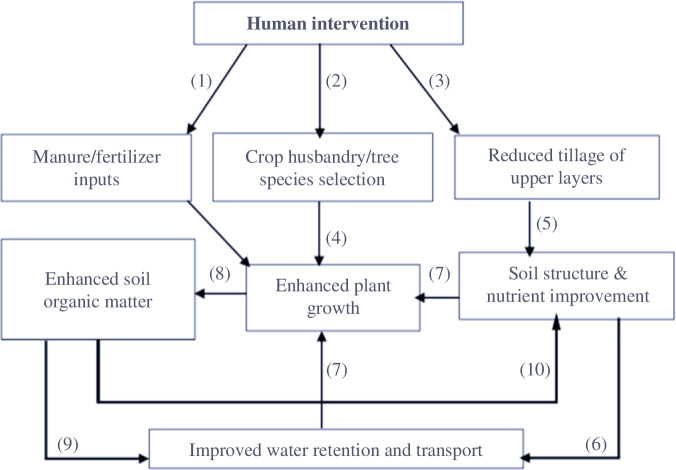
Simple schematic illustration of proposed mechanisms underlying forest-island creation.

Although the mechanisms of forest island formation in West Africa have been established [7], there is limited understanding of the biogeochemistry underpinning the phenomenon except qualitative descriptions of a few soil profiles of anthropogenically formed *Anogeissus* groves on abandoned village sites in Ghana [14]. We, thus, speculated that a variety of factors are involved, the net effect of which is a vastly improved nutrient and carbon contents compared to the surrounding savanna landscape (figure 1).

We envisaged the following mechanisms to be involved:

1. Considerable human and animal waste inputs resulting in enhanced availabilities of nutrients. In some cases, this may also be supplemented by synthetic fertilizer inputs, for example, NPK fertilizers being applied to food-cropped areas.
2. Species typically of forested areas are often selected, as are legumes, which may contribute nitrogen to the system.
3. Villagers typically till (reduced tillage) the upper 20 cm layer of soil.
4. Enhanced nutrient inputs are directly translated into increased levels of nutrients for plant growth, with leguminous species introduction also contributing.
5. Soil structure is improved through reduced tillage activities.
6. Upper layer soil structure improvement, results in improved water retention characteristics with high soil hydraulic conductivities.
7. The synergism arising from improved water and nutrient availabilities resulted in enhanced plant growth.
8. Overall, the increased productivity of the system is shown in greater organic carbon inputs.
9. Improved soil structural characteristics (e.g. aggregate stability) result in improved soil hydraulic properties. There thus exists a first feedback loop ((8)(9)(7)) linking plant productivity to soil structural characteristics mediated through soil carbon variations.
10. Enhanced soil (C) also results in higher cation exchange capacity. This then gives rise to a second positive feedback loop ((8)(10)(7)).

### 1.3. Objectives

In the attempt to explore the science underpinning the forest island phenomenon, we aimed to the following: (i) examine the soil mineralogical and chemical characteristics of the forest islands and adjacent natural savannas and croplands, (ii) assess soil aggregate stability and its drivers in forest islands and the adjacent ecosystem types in West Africa, and (iii) evaluate soil carbon, nitrogen and phosphorus dynamics of the forest islands. We also aimed to build and strengthen the research capacities of students and young scientists, respectively, in soil science and analytical skills in the African institutions in cutting-edge research.

#### 1.3.1. General research methodology

**Figure 2.**
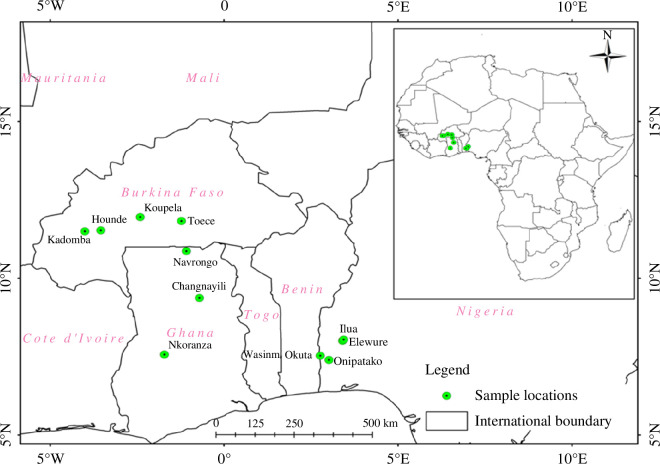
Location of study areas. *Source*: Bougma *et al*. [5].

**Figure 3.**
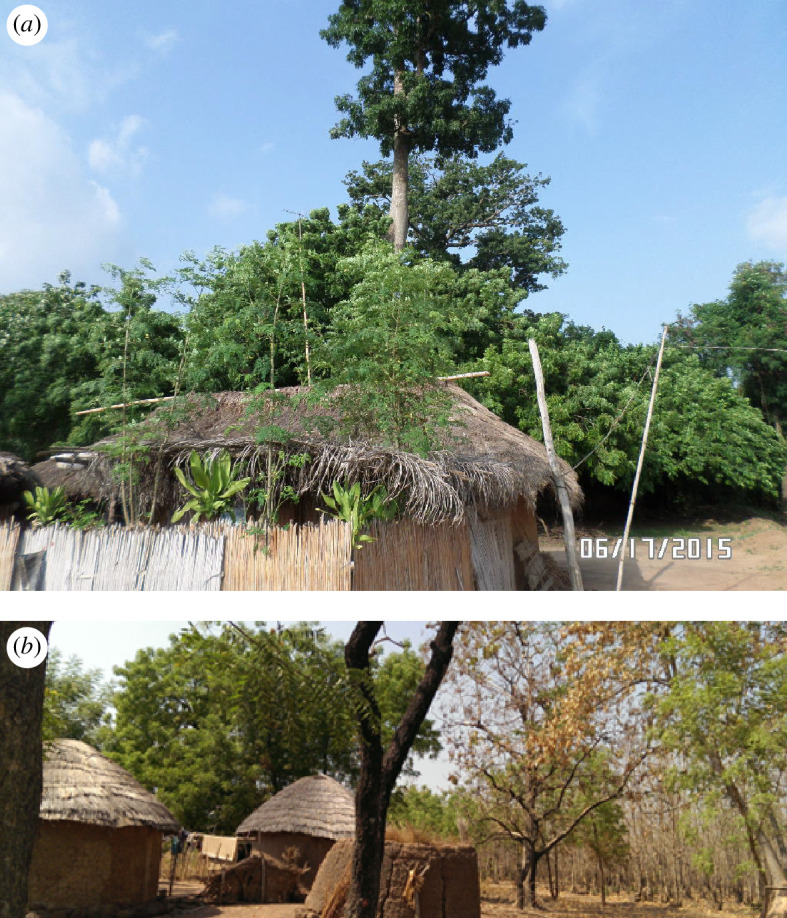
(*a*) Forest island in Devego, southern Ghana with adjacent village households. (*b*) Forest island in Nnorthern Ghana with adjacent village household *Source*: Mesele *et al*. [11].

Working across 11 sites (figure 2), three ecosystems namely forest islands (figure 3*a*,*b*), natural savannas and farmlands were identified and sampled from 2016 and 2017. At each location, the three land use types were selected and described as follows:

*Forest island* plots comprised tall trees, ranging between 15 and 20 m high with on average over 400 stems per hectare and with diameter at breast height (*D*) of > 10 cm. From site interaction with the villagers, most of these plots are known to have been preserved or protected for over 100 years.

*Savanna* plots were considered open natural vegetation type with tree density per hectare ranging from 50 to 100 (*D* > 10 cm). On average, these trees ranged from 5 to 10 m in height and were interspersed with abundant ground layers of grasses and herbs. These grasses are prone to periodic bush fires, especially in the dry season.

*Agricultural field plots or farmlands* which were under cultivation were selected in as close proximity as possible to the forest island and savanna plots (see Bougma *et al*. [5]). These in Burkina Faso were cotton or cereal-based fields and comprised monocultures of maize in Ghana and maize or intercrop of maize and cassava or legumes in Nigeria. The cropped fields received mostly mineral NPK fertilizer inputs from year to year mainly under natural rainfall regime. In most cases, the upper 0–20 cm layers are tilled continually.

## 2. Case studies

Working across three West African partner institutions namely the Kwame Nkrumah University of Science and Technology (KNUST), Ghana, Federal University of Agriculture Abeokuta (FUNAAB), Nigeria and the Institute of Environment and Agricultural Research (INERA), Burkina Faso in collaboration primarily with Imperial College London, we explored a number of case studies covering the major research themes.

### 2.1. Soil mineralogy and nutrient characteristics of the forest islands

Here, we investigated the mineralogy of the soils of the forest islands across West Africa in comparison with adjacent land use types namely natural savanna and agricultural lands. Soil chemical properties relating to soil fertility improvement were assessed and compared among the three land use types (see for more details Mesele *et al*. [11]). We aimed to gain insight into what extent forest islands are the products of major soil differences or whether their establishment was modulated by human-originated soil nutrient improvement. We, thus, aimed to provide a novel understanding of the mechanisms underlying the transformation of savanna areas around the homestead into luxuriant forest vegetation types through anthropogenic impacts on the landscape.

We studied soil mineralogy with X-ray diffraction (XRD) of the samples at the Natural History Museum, London using the fine earth fraction (< 2 mm) from depths > 50 cm. Soil exchangeable cations namely calcium, magnesium, potassium and sodium were determined on ICP-OES at the Federal University of Agriculture Abeokuta, Nigeria acquired through the Royal Society-FCDO (Foreign, Commonwealth and Development Office) award.

We observed similarities in the key mineralogical compositions of the forest islands and the adjacent croplands and savannas (table 1), pointing to the fact that soil mineralogy had no direct influence on the development of the unique West African forest islands [11]. As an example, the soils of all three ecosystem types of Changnayili (CHN) and Nkoranza (NKZ) in Ghana, Elewure (ELE) and Onipatako (ONP) in Nigeria, and Toece (TOE), Hounde (HOU), Kadomba (KAD) and Koupela (KPL) in Burkina Faso had dominant mineralogy of kaolinite and quartz. This provides further evidence of anthropogenic influence resulting in the formation of the forest islands. The established forest islands were protected from exploitation, encroachment and fire by the indigenous people. Similar cases of forest islands have been reported around local villages in south-eastern Guinea by Fairhead & Leach [6], which were created and maintained by villagers. However, the detailed mineralogy of such forest islands were not documented. Our work showed higher nutrient (nitrogen, phosphorus, potassium, etc.) status of the forest islands than the adjacent farmland and savanna (see [11]), implying improved soil conditions as a result of the anthropogenic impacts. We observed that soil organic matter and improved soil potassium from the anthropogenic activities played a dominant role in the formation of the forest islands demonstrating the importance of combined water retention and available potassium (CWAK) effects (Lloyd *et al*. [10,15]). Thus, we showed that ‘maintenance and preservation of such forests around homesteads exemplifies an age-long process of soil improvement in West Africa’ [11].

**Table 1.** Soil mineralogical profile of forest islands, adjacent farmlands and open savannas.

country code	sites	quartz	kaolinite	feldspars	mica/illite	goethite	haematite	variscite/phosphosiderite	chlorite/anatase	smectites	hornblende/boehmite/zeolites
NGA	ELE 01	+++	++	−	++	+	−	−	−	−	−
NGA	ELE 02	+++	+++	+	t	t		t	−	−	−
NGA	ELE 03	+++	+++		++	+	−	−	t	−	−
NGA	ILU 01	+++	+++	+++	+	++	+	−	−	−	
NGA	ILU 02	+++	+++	+++	−	t	−	−	−	−	H+
NGA	ILU 03	+++	+++	+++	+	+	+	−	−	−	H+
NGA	ONP 01	+++	+++	−	−	−	++	t	Ct		
NGA	ONP 02	+++	+++	−	−	−	++	−	−	−	−
NGA	ONP 03	+++	+++	−	−	−	++	−	−	++	−
NGA	WSM 01	+++	T	+++	t	−	−	−	−	−	H+, Zt
NGA	WSM 02	+++	T	+++	t	−	−	−	−	−	H++
NGA	WSM 03	+++	−	++	−	−	−	−	−	++	Zt, Ht
BFA	HOU 01	+++	+++	−	−	−	++	−	At	−	−
BFA	HOU 02	+++	+++	−	t	t	++	−	At	−	−
BFA	HOU 03	+++	+++	−	−	−	++	−	At	−	−
BFA	KAD 01	+++	+++	−	−	+	−	−	−	−	Zt
BFA	KAD 02	+++	++	−	−	t	−	−	−	−	Zt
BFA	KAD 03	+++	+++	−	−	+	−	−	−	−	Zt
BFA	KPL 01	+++	+++	+	−	+	−	t		−	Z+
BFA	KPL 02	+++	+++	−	−	+	−	t	−	t	−
BFA	KPL 03	+++	+++	+	−	+	−	t	−	−	Zt
BFA	TOE 01	+++	+++	++	+	−	−	−	−	−	−
BFA	TOE 02	+++	+++	+	−	−	−	t	−	−	−
BFA	TOE 03	+++	+++	−	++	−	−	−	−	−	−
GHA	CHN 01	+++	+	−	+	+	−	−	−	t	B+
GHA	CHN 02	+++	+	−	t	t		t	−	−	Bt
GHA	CHN 03	+++	+++	−	+	+	−	−	−	−	Bt
GHA	NAG 01	+	t	+++	+++	−	−	−	−	+++	Ht
GHA	NAG 02	+++	+	−	−	+	−	−	−	+++	Ht
GHA	NAG 03	+++	+	−	−	+	−	−	−	+++	Ht
GHA	NKZ 01	+++	+++	−	−	++	++	−	A+	−	−
GHA	NKZ 02	+++	+++	−	−	−	++	−	−	−	−
GHA	NKZ 03	+++	+++	−	−	−	++	−	−	−	−

*Source*: Mesele *et al*. [11].

+++, dominant; -, not detected; +, some/few; 01, forest island; 02, farmland; 03, savanna; ++, abundant; A+, anatase in few quantities; A, anatase; At, anatase in trace quantities; B+, boehmite in few quantities; B, boehmite; BFA, Burkina Faso; Bt, boehmite in trace quantities; C, chlorite; CHN, Changnayili; Ct, chlorite in trace quantities; ELE, Elewure; GHA, Ghana; H, hornblende; HOU, Hounde; ILU, Ilua; KAD, Kadomba ; KPL, Koupela (BNF); NAG, Navrongo; NGA, Nigeria; NKZ, Nkoranza; ONP, Onipatako; t, trace; TOE, Toece; WSM, Wasinm Okuta; Z, zeolites; Zt, zeolites in trace quantities.

### 2.2. Soil aggregate stability

Soil aggregate stability plays an important role in the reduction of soil erosion of the landscape and in enhancing soil hydrology. Our study of soil aggregate stability in Bougma *et al*. [5] contributed to the understanding of edaphic characteristics of forest islands in comparison with adjacent ecosystems. Considering earlier studies on the importance of vegetation cover on soil quality [16,17], we speculated that forest islands consisted of more water-stable soil aggregates than their surroundings.

Here, we sampled soils from each of the ecosystems at 0–5 and 5–10 cm depths (for details see Bougma *et al*. [5]). Soil aggregate stability was determined in three aggregate groups defined as ‘macroaggregates’ (4000–500 μm), ‘mesoaggregates’ (500–250 μm) and ‘microaggregates’ (250–53 μm) by the wet sieving method described by Mathieu & Pieltain [18]. The proportion of stable aggregates in each aggregate-size groups was determined.

**Figure 4.**
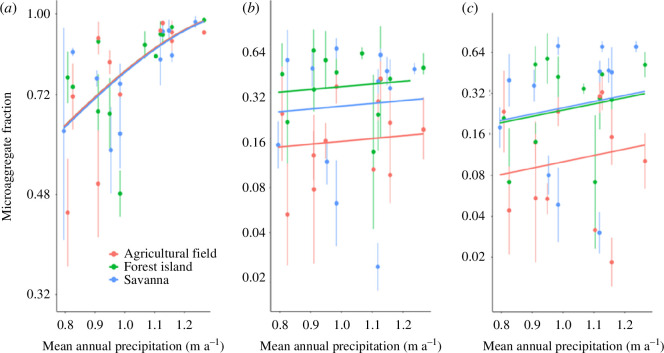
Effect of land use and mean annual precipitation on soil aggregate fractions at 0–5 cm depth. (*a*) Microaggregates; (*b*) mesoaggregates; (*c*) macroaggregates. Symbol and line colours are as indicated in panel (*a*), with the fitted lines representing the fixed component of the model fits. Bars represent standard deviations. *Source*: Bougma *et al*. [5]

We observed generally more stable meso-aggregates and macro-aggregates in the forest islands and savannas at both 0–5 and 5–10 cm depths than croplands across locations (see fig. 4 in Bougma *et al*. [5]) indicative of the negative impacts of continuous land cultivation on soil aggregate formation. The proportion of micro-aggregates increased strongly (*p *< 0.01) with mean precipitation amount (*P*_A_) but with no effect (*p > *0.1) of land use type evident (figure 4). However, for both the larger aggregate size groups (viz. meso-aggregates and macroaggregates), we found very different patterns of variation with there being no dependence of soil aggregate stability on *P*_A_ but with effects of land-use evident. The dependence of micro-aggregate stability on precipitation amount (figure 4) is consistent with earlier studies [19–21] reporting increased aggregate stability with precipitation. Presumably, this observation was a result of the widely reported influence of increased precipitation on soil microbial biomass [22–25], which plays a key role as binding agent in soil aggregation [26].

Based on our findings, we highlight that soil microaggregate formation in the West African ecosystems was climate (precipitation) driven whereas meso- and macro-aggregates were land-use driven, the latter situation mainly owing to vegetation cover in the denser forest islands and the natural savanna characterized by higher carbon inputs than adjacent agricultural lands.

### 2.3. Distribution of soil carbon and nitrogen in forest islands

Here, we provided an understanding of the biogeochemical mechanism underlying the formation of forest islands and savannas in West Africa. This is interesting as both forest islands and savannas were found at the same localities with similar climatic conditions, thus, eliminating climatic factors (e.g. climate) of vegetation formation.

Soil organic carbon (SOC) and nitrogen are more likely to be influenced by vegetation and are considered major indicators of soil fertility [27]. Hence, studying how they vary with other soil parameters under anthropogenic influence may give better insights into how the forest islands are formed. In our study, we aimed to provide a focus on local factors driving the balance between savannas and forest islands.

Soil sampling was performed at 0–5 and 0–30 cm depths across all 11 locations in West Africa. The SOC and total nitrogen content were measured using the Elementar analyser (Vario MACRO cube, Elementar Germany) at the Kwame Nkrumah University of Science and Technology, Ghana acquired through the Royal Society-FCDO award. To confirm the general notion that inorganic carbon content in tropical soils is negligible, few samples with pH ≥ 6.5 were pre-treated with HCl [28] before SOC measurement but with no significant differences recorded between the HCl-treated and the non-treated soils.

We observed soil carbon concentrations in the ecosystems to range from 0.29% in Toece farmland, Burkina Faso to 8.96% in Navrongo forest island in Ghana at the 0–5 cm depth, and 0.27% in Kadomba farmland in Burkina Faso to 4.64% in Navrongo forest island at the 0–30 cm depth in a general increasing order of farmland < Savanna < forest island (results not shown) at each site. Soil total nitrogen ranged from 0.34% in forest island at Navrongo in Ghana to 0.03% in farmland at Wasinm Okuta and Elewure in Nigeria at 0–30 cm depth. At both depths, the two parameters were markedly higher in the forest islands than both the savanna and farmland soils due possibly to anthropogenic soil improvement and enhanced woody plant growth with higher organic inputs. The decrease in SOC (and nitrogen) concentrations in cultivated soils is mainly linked to intensified cropping system practices such as crop residues removal, inadequate organic substrates addition [29] and continuous tillage practices. Conventional tillage practices cause inversion of soil layers which exposes soil organic carbon to decomposition [30], leading to less accrual over time in agricultural lands. The results of this study clearly demonstrates forest islands as greater sinks of carbon and nitrogen through improvement in nutrient cycling mechanisms than adjacent natural savanna and agricultural lands. This has implications for overall soil health and ecosystem sustainability in the long term.

### 2.4. Persistence of soil organic carbon in *Anogeissus* grove/forest island in Mole

As part of efforts to understand carbon and nutrient cycling in the anthropogenically formed forest islands, we revisited a unique plot of an abandoned forest island (*Anogeissus* grove) in the Mole National Park of Ghana, which was first studied by Sobey in 1974 [14].

The Mole National Park of Ghana located in the Guinea savanna agroecological (i.e. tropical continental climatic) zone of Ghana was established in 1958 [31]. At the time, a number of human settlements were removed. These settlements had established forest islands comprising mainly *Anogeissus leiocarpus* (i.e. African birch belonging to the family Combretaceae) and management of these forests stopped. One such forest island within the Park was studied in 1974 in detail for its vegetation and some soil parameters [14] with higher nutrient status than the adjoining savanna, which formed the basis of the SOFIIA (Soils of Forest Islands in Africa) research. The *A. leiocarpus* is known for its medicinal value and has many other uses including as rafters and poles for building traditional houses (information obtained from local carpenters)

We revisited the site 42 years after the previous survey was carried out and 58 years after the original human activities stopped to understand persistence of soil traits after abandonment and to quantify the soil carbon sequestration potential of the abandoned grove. We hypothesized that ‘human-induced soil improvement can persist in savanna landscape over decadal timescale and modulate soil carbon sequestration in the long term’ (see Logah *et al*. [32]). Here, we present key results on the mineral-associated organic carbon (MAOC) and particulate organic carbon (POC) pools in comparison with adjacent natural savanna vegetation. We also show the amount of carbon and nitrogen stabilized in each of the pools measured.

The POC and carbon associated with the silt+clay matrix (MAOC) reported herein were separated using the modified granulometric method of Cambardella & Elliot [33]. The carbon and nitrogen contents of each pool were determined using the Elementar analyser (Vario MACRO Cube, Germany).

**Figure 5.**
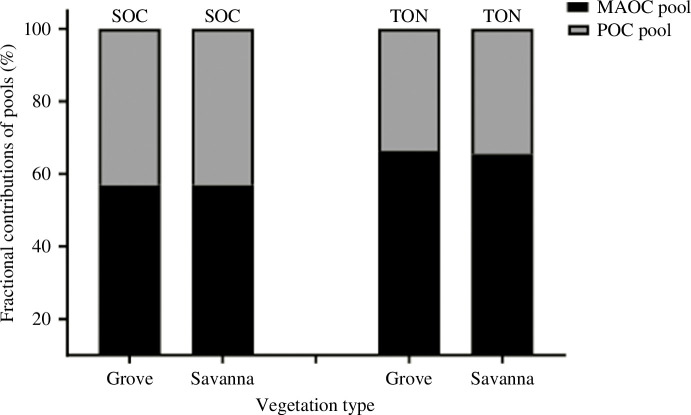
Proportions of particulate organic carbon (POC) and mineral associated organic carbon (MAOC) in soil organic carbon (SOC) and soil total N (TON). *Source*: Logah *et al*. [32]. abbrevaitions: POC, Particulate organic carbon; MAOC, Mineral associated organic carbon; TON, Total soil organic nitrogen; SOC, Soil organic carbon.

The POC and MAOC with varying degrees of persistence [31,34] differed significantly between the grove and the savanna (table 2), showing the extent to which anthropogenic soil improvement through local efforts with the grove can contribute to soil carbon sequestration in the longer term after abandonment. The grove’s greater POC than that of the savanna (table 2), reflects its ability to accumulate from leaf litter and necromass, more decomposable substrate [35]. We observed greater carbon stabilized in the mineral fraction (*ca* 55%) in both the grove and the savanna than the particulate fraction (figure 5) as found elsewhere (e.g. [36,37]). Thus, ‘our results suggest that the greater portion of soil carbon sequestered in the anthropogenic grove is persistent’ [32]. By virtue of its persistence in soil [38], global efforts are directed towards increasing carbon sequestration through the MAOC pool. Overall, our study clearly indicates that sustainable land management through indigenous effort in West Africa aligns with the recent global initiative of ‘re-carbonizing global soils’ [39], serving unarguably as a right step towards achievement of the ‘4p1000’ initiative towards ecosystem services and sustainability [32,40].

**Table 2.** The POC and MAOC of *Anogeissus* grove and savanna at Mole.

	vegetation type		
carbon pool (g C kg^−1^ soil)	grove	savanna	***p-*value**
POC	5.94 ± 0.56a	3.37 ± 0.40b	0.05
MAOC	11.65 ± 0.19a	8.00 ± 0.17b	0.04

Values followed by different letters in row are significantly different at 5%; Adapted from Logah *et al*. [32].

±, standard deviation; MOAC, mineral associated organic carbon; POC, particulate organic carbon.

## 3. Successes achieved in capacity strengthening and networking

The SOFIIA consortium was a North–South–South collaboration between Imperial College London (ICL), Kwame Nkrumah University of Science and Technology (KNUST), Ghana, Federal University of Agriculture Abeokuta, Nigeria, Institute of Environment and Agricultural Research (INERA), Burkina Faso with the Wageningen University, Netherlands also joining in. The strength of the collaborations between the partner institutions culminated in the training of three primary PhD students in Ghana, Nigeria and Burkina Faso who are now contributing to science in Africa and beyond.

### 3.1. PhD students trained

Primarily, the three PhD students competitively selected and trained under the research collaboration are:

— Caleb Melenya at the Kwame Nkrumah University of Science and Technology, Ghana: Caleb was enrolled in 2015 and successfully graduated in 2020, having worked on ‘Soil carbon, nitrogen and phosphorus dynamics of forest islands and adjacent ecosystems in West Africa’. He served a 2-year postdoctoral term and as a Department Associate at the Hungarian University of Agriculture and Life Sciences, Hungary where he contributed significantly to teaching and research including soil carbon projects namely Soils4Africa, EJP Soils C-arouNd, etc. He is now employed with the CSIR-Crops Research Institute, Fumesua, Kumasi as a Research Scientist.
— Samuel Ayodele Mesele was enrolled under the project at the Federal University of Agriculture Abeokuta, Nigeria in 2015. He successfully completed his PhD in 2021 after having worked on ‘Chemical, mineralogical characterization and quality potential of soils of forest islands in some selected savanna ecology of West Africa’. Dr Mesele now works with the International Institute of Tropical Agriculture (IITA) in Nigeria and is currently part of the team leading the field campaign of the Soils4Africa project funded by EU under the Horizon2020 program aimed at developing soil information system in Africa.
— Amelie Baomalgre Bougma of INERA, Burkina Faso enrolled under the collaboration at the Polytechnic University of Bobo-Dioulasso, Burkina Faso in 2015. Her study focused on ‘Soil hydro-physical properties of forest islands’. She continues to work with INERA.

In addition to the three primary PhD students, the collaboration saw to the successful training of 11 other postgraduate students affiliated to the project across the three African Institutions. These include one PhD in Ghana and 10 master's students from all three West African countries all of whom had successfully completed their research. These comprised a combination of male and female students. Apart from their mainstream training supported by the collaboration, these students also benefited from research skills training through capacity-building workshops organized by the SOFIIA consortium.

### 3.2. Networking or some new research collaborations established

The strength of this collaboration was also seen in further networking and successful grant applications viz. the SABMiller Royal Society exchange programme between KNUST and ICL in collaboration with University of Aberdeen, Scotland and the Natural History Museum, London; the European Joint Program (EJP Soil) C-arouNd project between KNUST and 12 partner Institutions across Europe, South America, Africa and Oceania. The C-arouNd project is largely a continuity of the Royal Society-FCDO Africa Capacity Building Initiative (ACBI) in Ghana, which seeks to build a long-term database on soil carbon and soil properties of the forest island or African dark earth sites.

The social aspects of the Royal Society-FCDO funded SOFIIA collaboration comprised community engagement in tree planting activities in local communities of some of the study sites in Burkina Faso, Ghana and Nigeria.

### 3.3. Instrumentations and skills training

The award supported the African partner institutions through the acquisition of many pieces of scientific laboratory equipment including the throughput CN Elemental analyser Vario MACRO cube (Elementar, Germany) at KNUST, ICP-OES (Thermo Fisher Scientific) at FUNAAB and Neutron probe at INERA. Altogether, 55 pieces of equipment were acquired, which have since enhanced state-of-the-art research in the West African countries. Six capacity-building workshops were successfully organized in science communication, trace gases, R statistics, soil fractionation, among others in the African Institutions, which built the capacities of many postgraduate students, strengthening also the capacity of laboratory technicians and some academic staff. The three primary PhD students were supported to attend international conferences such as the 6th International Symposium on Soil Organic Matter in Rothamsted Research in 2017 in Harpenden, UK; 5th iLEAPS Science Conference in 2017 in Oxford; the 21st World Congress of Soil Science in Rio, Brazil in 2018 where they presented some of their research findings.

**Figure 6.**
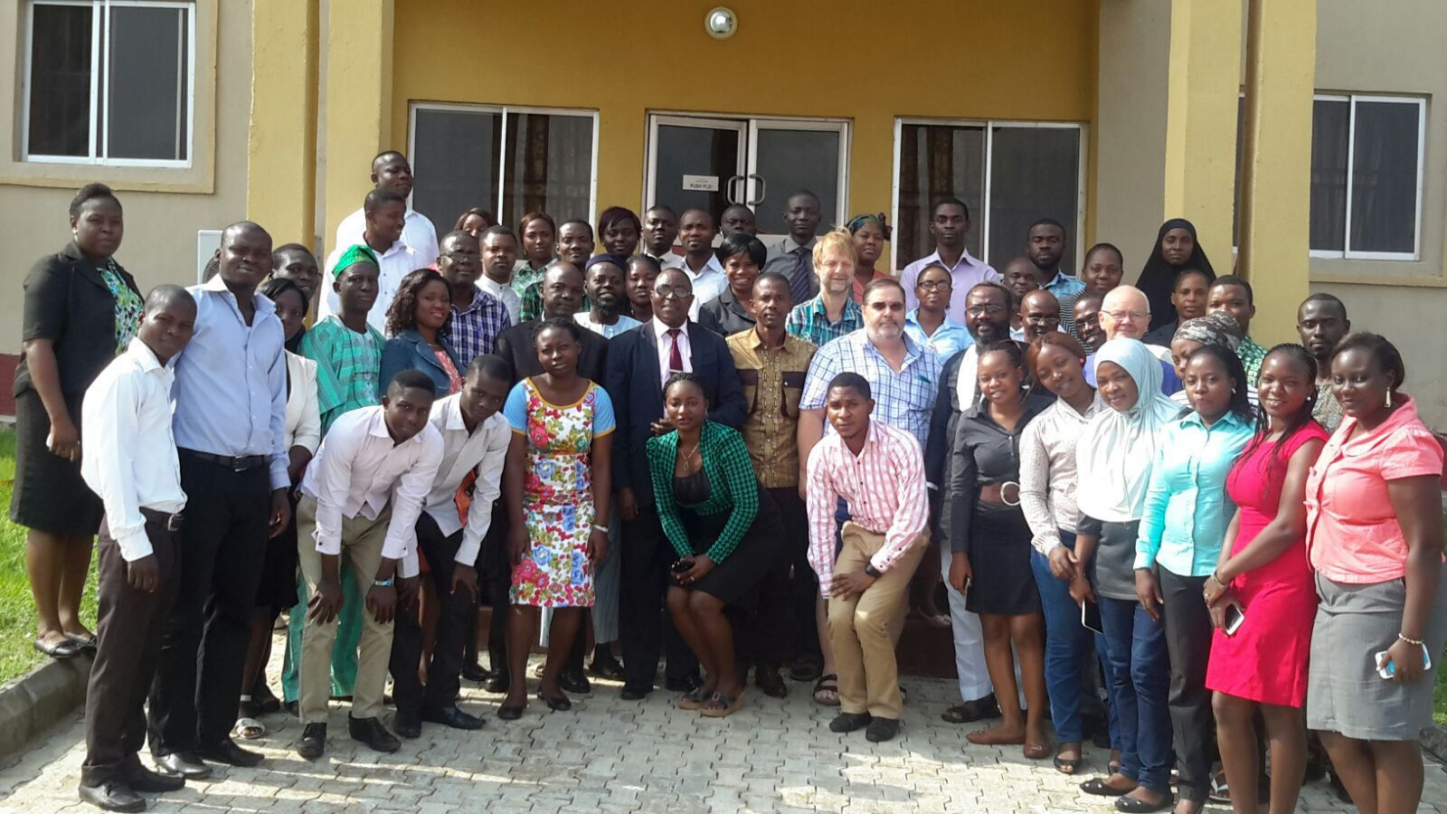
SOFIIA Consortium capacity -building workshop on science communication in FUNAAB, Nigeria.

Overall, the research collaboration over the 7-year period (by extension) has thus had significant success in capacity building or strengthening of postgraduate students and staff of the African institutions in Ghana, Nigeria and Burkina Faso (e.g. see figure 6).

## 4. Conclusion

We conclude that the North–South–South research collaboration has been successful in exploring the science underpinning the West African forest island phenomenon and in capacity building of young scientists across the West African institutions involved. The work has established that soil mineralogy itself plays no direct role in the formation of forest islands, which had enriched carbon and soil nutrients status than the surrounding ecosystems. Soil micro-aggregation in the West African ecosystems was climate (precipitation) driven whereas meso- and macro-aggregations were land-use driven. We highlight the importance of forest islands in providing a better sink for soil carbon sequestration than natural savannas with positive implications for the ‘4p1000’ initiative for climate change mitigation in West Africa. The young scientists trained during the collaboration are remarkably driving cutting-edge research in the sub-region and beyond.

## Data Availability

Table 1 was sourced from publication of the authors’ paper in *Plant and Soil* and Figures 3-5 were also from the authors own publications (see [5,11,32]).
